# Disruption and recovery: a qualitative study on health services for vulnerable populations during the pandemic

**DOI:** 10.3389/fpubh.2026.1744878

**Published:** 2026-02-23

**Authors:** Harimadhav Viswanathan, Pankaja Raghav, Prem Prakash Sharma, Pritish Baskaran, Mukul Maheshwari, S. R. Aswathy, Mukund Gupta, Vishal Mehrara

**Affiliations:** Department of Community Medicine and Family Medicine, All India Institute of Medical Sciences Jodhpur, Jodhpur, India

**Keywords:** access to healthcare, catch-up, health equity, health service disruption, pandemic, vulnerable groups

## Abstract

Public health emergencies like pandemics results in systemic disruptions, leading to substantial indirect consequences beyond direct morbidity and mortality. The experiences from previous outbreaks have shown disproportionate effects on the vulnerable groups. This study aims to identify the challenges faced by these groups in attaining routine health services during the COVID-19 pandemic and the strategies used for service recovery. A qualitative study done in Jodhpur district in Western India using inductive thematic approach. Data were collected through eight key informant interviews with healthcare workers and twenty in-depth interviews with patients from vulnerable groups. Vulnerable populations reported reduced healthcare access due to fear, transport barriers, and staff shortages, often resorting to private care. Frontline health workers faced initial service disruptions but later resumed community outreach with COVID-appropriate behavior. Health facilities adapted to the pandemic by prioritizing essential services, utilizing teleconsultations, and ensuring home delivery of medications despite PPE shortages and workforce redistribution. Vaccination faced challenges including digital registration issues, misinformation, and hesitancy, mitigated through local leadership and community engagement. Catch-up programs effectively addressed missed immunizations and TB care, highlighting system resilience and recovery efforts. Vulnerable groups have specific and increased needs during times of crises like pandemics, that must be addressed specifically. Targeted interventions that are acceptable and appropriate should be implemented to bridge gaps in healthcare access. Future preparedness plans must institutionalize adaptive strategies to ensure equitable access to care and safeguard the vulnerable populations during crises.

## 1 Introduction

Epidemics of emerging and re-emerging illnesses have been on the rise since the turn of the 21st century. The world faced a global health crisis unlike any in the past 100 years in the form of COVID-19. It took the world by a storm- killing people, spreading human suffering, and upending people's lives. As per the WHO COVID situation reports of 05 October 2025, over 779 million confirmed cases and over 7.1 million deaths have been reported globally (1). A total of 45 million covid cases and 5,33,644 deaths were reported in India till December 2024. Rajasthan had the eighth highest burden amongst 29 states and 7 union territories in India with 13,27,819 cases reported and 9743 deaths (2). Within Rajasthan, Jodhpur was the second most affected district, after the state capital Jaipur (3).

Besides causing widespread disease and death, COVID-19 also possesses a significant risk of indirect morbidity and mortality due to disruption in the management of other preventable and treatable diseases (4). The aftermath of the Ebola outbreak in West Africa illustrated that the indirect effects of such epidemics often outweigh their direct impacts, further straining already burdened health systems (5). Access to healthcare for non-pandemic health issues decreases due to a mix of demand and supply chain disruptions. The most mentioned causes for service disruptions include cancellation of elective care, closure of population-level screening programmes, government transportation lockdown, and shift of staff to provide emergency relief activities (6). The COVID-19 pandemic was no different and had an inequitable and disproportionate impact on vulnerable populations, reversing decades of progress toward healthy population.

Vulnerability can be defined as the physical, social, economic, and environmental factors that increase the susceptibility of an individual or a community to the impact of hazards. Vulnerable population or the population at high risk include older adult, children under-5 years of age, pregnant women, individuals with underlying chronic health conditions (e.g., diabetes, heart disease, and respiratory illnesses), or immunocompromised people. These groups often have weakened immune systems or pre-existing health conditions that make them more susceptible to severe outcomes if infected (7). During public health crises, they experience far more pronounced negative health and socioeconomic impacts than the general population. The impact is also severe on the lower socioeconomic groups due to the pre-existing wealth inequality and social exclusion, which limit their access to essential healthcare services and resources (8).

Despite the evident challenges faced by vulnerable populations, there remains a paucity of systematic qualitative evidence examining the challenges in attaining health services, targeted mitigation strategies, and recovery process in an integrated manner (8). The existing literature is predominantly quantitative and often fail to capture the context-specific dynamics. Most of the existing studies have explored disruptions in routine healthcare, COVID-19 vaccination, and pandemic wave specific dynamics in isolation, limiting a comprehensive understanding of how these interconnected factors collectively influenced the health service delivery for the vulnerable groups. This study aims to bridge these gaps by understanding the challenges faced by people with health vulnerability in assessing the routine healthcare services and COVID vaccination through a qualitative approach. It also aims to identify the strategies adopted by patients and health system to overcome these challenges and facilitate service recovery in the post-pandemic period.

## 2 Methodology

### 2.1 Study setting

This qualitative study was a part of a bigger mixed-methods study aiming to assess the epidemic preparedness of health facilities and the impact of COVID-19 pandemic in Jodhpur district, Rajasthan. The study was undertaken in the primary and secondary level government health care facilities of Jodhpur district. Primary level health care facilities included Primary Health Centers (PHCs), while secondary level facilities include Community Health Centers (CHCs), Satellite Hospitals (SHs) and District Hospitals (DHs).

### 2.2 Sampling procedure

For the qualitative component of the study, data was collected through key informant interviews (KIIs) and in-depth-interviews (IDIs). Purposive sampling was used to select the participants for the interviews. This non-random sampling approach involves the identification and selection of individuals who are knowledgeable about and have direct experience with the phenomenon under investigation (9). Maximum variation strategy was used to capture a diverse perspective from different levels of healthcare facilities, healthcare workers, and vulnerable population groups (9).

One PHC, CHC, satellite hospital and district hospital were selected from the healthcare facilities in the district. Health facilities were selected to represent primary and secondary levels of care, as these facilities are central to the delivery of essential services for vulnerable populations and allowed exploration of service disruptions and adaptations across the referral continuum.

For the KIIs, Medical officer in charge of the selected healthcare facilities and one of the Accredited Social Health Activist (ASHAs) from each facility were interviewed. From patients attending these health care facilities, those belonging to vulnerable groups like pregnant women, elderly people (above the age of 60 years), malnourished people, mother of under-five children, and people who were immunocompromised were interviewed for the IDIs. Participants were purposively selected to include both service providers and service beneficiaries, enabling exploration of supply-side and demand-side perspectives on healthcare delivery.

Eight healthcare workers were interviewed, while patients interviews were conducted until data saturation was reached. Data saturation was considered when additional data collection did not yield any new themes, categories, or conceptual insights (10). In the present study, thematic repetition started after 14–15 interviews and saturation was considered complete after 20 patient interviews. So, a total of 28 interviews were conducted (eight KIIs with healthcare workers and 20 IDIs with patients).

### 2.3 Data collection procedures

Semi-structured interview guides were developed separately for healthcare providers and patients based on an extensive review of existing literature and detailed discussions among the authors. Semi-structured interview guides allow flexible collection of in-depth information while remaining aligned with the study objectives (11). A review of existing literature helped in developing a comprehensive understanding of the topic and identification of areas where additional empirical data were needed (12).

The healthcare providers were asked about the routine services provided before and during the pandemic, challenges faced in providing service during the pandemic, surveillance activities in the area, the status of services post lockdown and details regarding any catch-up programmes. While the patients were asked questions on the ground of the difficulties they faced in accessing the treatment during the pandemic, apart from the questions on the usual services they were getting from the health facility. Specific questions on COVID-19 vaccination were also incorporated to get an understanding of the real challenges faced in obtaining the vaccine.

The interview guides were internally tested through mock interviews conducted within the research team to assess the clarity, flow, and alignment of the contents with study objectives. It also helped to identify ambiguities, leading questions, and potential interviewer bias. Based on this process, the wording and sequencing of the questions were modified prior to data collection (11). All the authors underwent formal training in qualitative research through national level workshops ranging from three to five days duration.

Interviews were conducted between November 2021 and April 2022 (6 months). All interviews were conducted in the respective health facilities. They were conducted in a quiet and private space, with only the interviewer and participant present. This ensured privacy and confidentiality thereby encouraging participants to express their experiences openly and honestly. Informed signed consents were obtained prior to the interview. All the interviews were conducted in the local language- Hindi and voice recorded using a mobile phone.

## 3 Data analysis

### 3.1 Analytical approach

An inductive thematic approach was used to analyze the data. It involves the development of themes directly from the data without applying any pre-existing codes or theories, thereby allowing the findings to be grounded in participants' experiences (13). This approach was appropriate for the study as it enabled an open and unbiased exploration of experiences related to healthcare delivery during the COVID-19 pandemic.

### 3.2 Coding and theme development

The audio recordings were transcribed into Hindi on the same day to avoid the loss of any vital information. This was done by the interviewer and another author proficient in Hindi. The transcript was then translated to English by a third author and then back-translated into Hindi by a fourth author. During translation, particular attention was paid to preserving meaning, tone, and culturally embedded expressions, prioritizing conceptual equivalence over literal translation. Any differences were resolved through discussion among the authors. This helped in ensuring linguistic accuracy and authenticity of the data collected.

The English transcript was used for data analysis. The analysis was done manually. The first step was familiarization with the transcripts through repeated reading. The transcripts were then subjected to line-by-line thematic analysis by two authors independently. This approach allowed close engagement with the data and is recommended for narrative qualitative studies with limited sample size (14). Verbatims were identified and initial coding was done to generate the detailed codes from the transcripts. The initial code lists generated by both authors were compared and discussed to reach to final agreement.

This was followed by an intermediate coding process in which conceptually similar codes were brought together and used for the development of sub-themes and more abstract categories. These categories were further examined and refined through advanced coding, wherein relationships between categories were explored and integrated to identify overarching themes. This structured, sequential process enhances the rigor and trustworthiness of the themes developed from the collected qualitative data (13, 15).

Data collection and analysis proceeded concurrently. Any discrepancies in coding or interpretation were resolved through dialogue and consensus among the researchers to enhance inter coder reliability (16). The final findings were summarized using tables and figures.

## 4 Rigor

The criteria proposed by Lincoln and Guba were used to ensure the rigor and trustworthiness of the study (17). Credibility was enhanced through purposive sampling with maximum variation, inclusion of both healthcare providers and service beneficiaries, independent coding by two researchers and consensus discussions. The detailed description of study setting, participants, and context adds transferability. While the use of a semi structured interview guide and sequential thematic approach had ensured dependability. Confirmability was strengthened by grounding findings in verbatim participant accounts and resolving interpretive differences through dialogue, thereby minimizing researcher bias.

## 5 Results

The 28 participants included four medical-officer-in-charge, four ASHAs, two pregnant women, two elderly individuals, five mothers of under-five children, four patients with diabetes mellitus (DM), five patients with hypertension (HTN), and two patients with tuberculosis (TB) (Table 1).

**Table 1:** Theme 1-challenges faced and mitigation strategies adopted in accessing routine health services.

**Verbatims**	**Codes**	**Subthemes**	**Themes**
Main difficulty was to travel this far	Traveling issues		
We had to get ourselves tested before getting admitted	Getting tested for admissions		
Fear of getting COVID on going to hospital	Anxiety	Challenges to meet healthcare needs	Challenges faced and mitigation strategies adopted in accessing routine health services
Had to pay for consultation as well for the medicine	Financial		
Initially there was a shortage in the masks, gloves, and the PPEs	Lack of protective measures		
Few of the staff was transferred to district hospital during the peak	Human resources shortage	Challenges in providing services	
Laboratory was closed during the COVID peak	Lab closure		
General OPD was not entertained Admissions restricted to ILI cases only	Diversion to COVID care		
The DOTS staff was keeping in touch with the patient and ensuring the regular supply of medicines	Ensuring continuity	Mitigation Strategies	
Drugs were distributed at their homes in regular intervals	Home delivery of drugs		
We had started tele consultation services	Tele medicine		
Strict covid guidelines followed in the hospital premises Physical examination was avoided unless necessary	COVID appropriate behavior		

Participants from vulnerable groups reported decreased visits to healthcare facilities. They faced numerous challenges in attaining healthcare like difficulty in transportation, fear of getting COVID-19 while going to hospitals and had concerns over lab closure and staff shortages. Some resorted to seeking care from private physicians due to limited access to government healthcare services, resulting in financial strain. Participants with chronic NCDs continued their daily medications despite the absence of routine consultations. Immunization services were disrupted for several months, and accessing routine laboratory investigations became difficult due to staff shortages and transportation issues.

ASHA revealed significant disruptions in their routine activities during the initial phase of the COVID-19 pandemic, particularly due to shortage of masks and sanitizer. Initially, all services were halted, including home visits, which are integral to their roles. As protective measures became available, ASHAs resumed their duties, adhering to COVID-appropriate behavior such as wearing masks, using sanitizers, and maintaining social distancing during home visits. They also emphasized the importance of cleanliness and changing clothes upon returning home after their fieldwork. Additionally, ASHAs provided counseling to community members regarding COVID-19 prevention and management. Healthcare workers demonstrated resilience, providing essential services without signs of stigma or fear.

**Figure 1:**
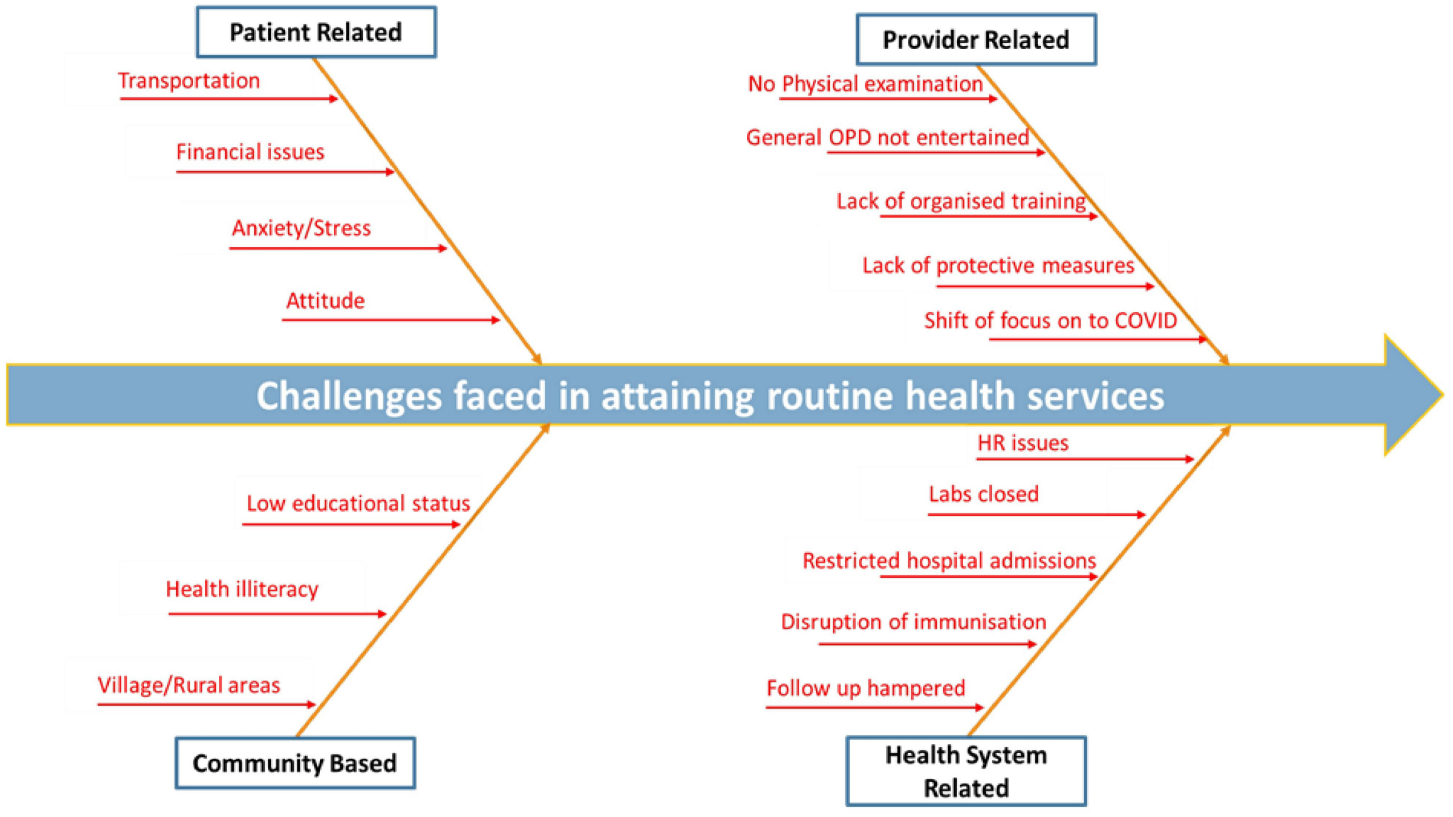
Fishbone diagram-challenges faced in availing routine health services.

Health facilities implemented COVID appropriate behavior including social distancing and adopted various measures such as limiting patient examinations to essential cases and suspending general outpatient services, admitting only those with influenza-like illness to inpatient departments. Routine laboratory services were temporarily halted, but efforts were made to conduct RT-PCR testing for COVID-19. Despite shortages of personal protective equipment (PPE), facilities managed staff effectively, transferring some to district hospitals during COVID peak. Collectively, these operational shifts significantly disrupted the routine health service delivery. The health system responded by shifting to telemedicine and home delivery of medicines for chronic non communicable diseases and tuberculosis (Figure 1).

Key informant interviews with healthcare workers revealed significant challenges in the vaccination process including difficulties in online registration in the rural setting and lack of proper identity cards among villagers (Table 2). Patients had encountered challenges, including difficulties in securing vaccination slots online, being charged by agents, and experiencing long waiting periods. E-Mitra agents played a crucial role in assisting with registration, and spot booking was implemented to streamline the process. But many of the E-Mitra agents were charging people heavily for slot booking and this imposed an additional financial burden on the people already struggling with the pandemic.

**Table 2:** Theme 2-challenges in COVID Vaccination.

**Verbatims**	**Codes**	**Subthemes**	**Themes**
Online registration was a bit complicated process it was difficult for the village people	Online mode	Registratio	Challenges in COVID vaccination
People are not having proper identity cards	Identity card		
Slots were getting filled very fast	Less slots		
E-mitra agent charged me Rs. 50 for one slot	Financial issues		
Problems due to lack of doses	Decreased availability	Availability	
Had to wait for more than 1 h to get vaccine	Long waiting period		
We got help from police to have control over the crowd	Huge crowd		
Never had issues in getting required doses on time	Rural-urban difference		
vaccine has lot of side effects Afraid of the side effects of the vaccination It might kill people	Afraid of side effects	Hesitancy	
Many who took vaccination were also getting COVID	Disease in vaccinated		
There were no cases in our area We are not going outside the village to Jodhpur city	Complacency		
If we take the vaccine we might die	Spread of false information		

Vaccination camps were organized in various villages, and police intervention was required for crowd control. Dedicated staff were deployed for on-site registration, and crowd management required additional support from police in these sites. Home-based vaccination was facilitated for bedridden patients. Urban hospitals had better vaccine availability compared to rural facilities.

**Figure 2:**
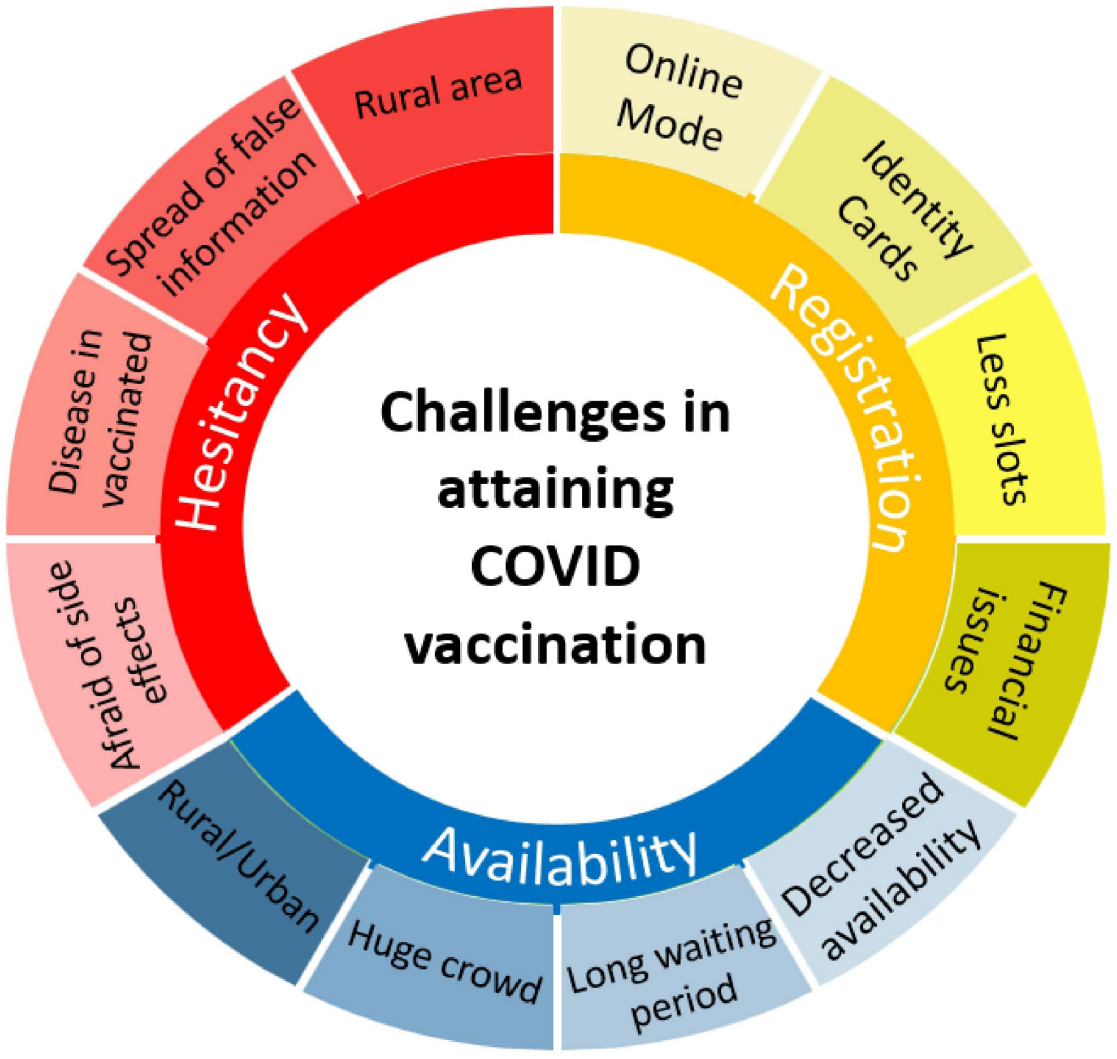
Challenges in attaining COVID vaccination.

Initially, villagers had fears about vaccination, citing concerns over side effects and misinformation. However, local leaders played a pivotal role in removing these fears and encouraging vaccination. Over time, more villagers came forward for vaccination, particularly after the second wave. Hesitancy was addressed through doctor-led counseling sessions, successful vaccination experiences, and community outreach efforts although misconceptions about vaccine safety and effectiveness persisted among some individuals. Despite these challenges, successful vaccination coverage was achieved through community efforts and effective communication strategies (Figure 2).

Patients from vulnerable groups reported differences between the two waves (Table 3). There was a decreased sense of fear during the second wave, leading to increased movement outside their homes for work and other activities. While adherence to safety measures was less strict compared to the first wave, the restart of bus services and widespread self-medication practices catalyzed the normalization of daily life activities despite the ongoing pandemic. They also reported receiving more thorough examinations, including checks for blood pressure and sugar levels in the health facilities.

**Table 3:** Theme 3-difference in healthcare services between two waves of COVID-19.

**Verbatims**	**Codes**	**Subthemes**	**Themes**
Less number of cases and deaths were reported in the first wave Second wave more fast spreading and more severe	Increase in second wave	Severity	Difference between two waves of COVID 19
More number of people used to go out of their houses	Increased outdoor activity	Community behavior and risk perception	
People were not afraid of the disease	Reduced fear of COVID-19		
Did not follow the rules as strictly as in the first wave	Decreased compliance		
More prepared in the second wave	Better preparedness	System readiness and resilience	
Bus services had restarted	Transportation		
Lab services were as before	Labs reopened	Restoration of routine services	
Physical examination was done as before Started to check bp and sugar	Physical examination restarted		

There was an evident disparity in the healthcare delivery during the first and second waves of COVID-19. During the first wave, fewer cases and deaths were reported, but people were afraid and adhered to safety protocols such as wearing masks. Medical officers and healthcare workers noted that individuals strictly followed preventive measures during this period. However, as the pandemic progressed and individuals became more accustomed to the virus, there was a sense of complacency and reluctance to follow government protocols.

During the second wave, the virus spread more rapidly and the symptoms were more severe. Despite challenges in convincing people to adhere to preventive measures, the overall situation improved as more individuals received the first dose of the COVID vaccine, and medical facilities became more prepared to handle the influx of cases. Laboratory services resumed and physical examinations were conducted as before during the second wave, indicating a shift in attitudes toward the virus and healthcare services.

**Figure 3:**
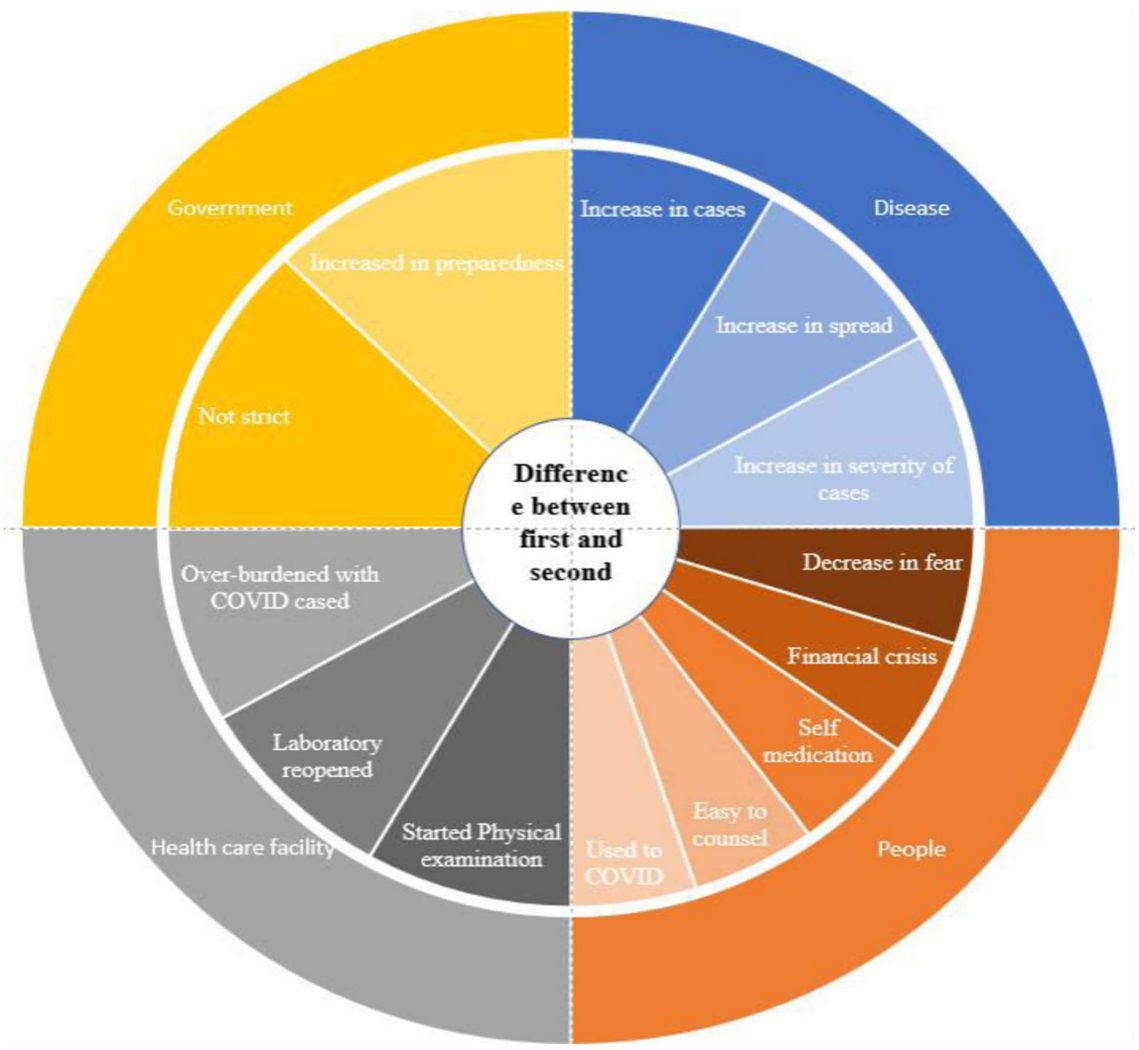
Difference between the two waves of COVID-19.

These findings underscore the evolving nature of the COVID-19 pandemic and the shifting attitudes of health system and communities between the two waves (Figure 3).

Patients from vulnerable groups noted the closure of Anganwadis but highlighted regular home visits by healthcare workers to deliver services, indicating efforts to resume routine activities (Table 4). Many of the participants reported that there was a sense of relief as healthcare facilities resumed operations. However, some patients expressed disappointment with the lack of follow-up from hospitals, although they appreciated efforts to provide missed services through catch-up programs.

**Table 4:** Theme 4-catch up programs.

**Verbatims**	**Codes**	**Subthemes**	**Themes**
Immunization catch-up was done	backlog addressed	Immunization	Catch-up programs
Cross checked the list and given vaccination	Cross checking		
Called and given the missed dose at the hospital	Catch up sessions		
TB patients were traced back	Continuity of care	Tuberculosis	
Anganwadis are still closed but they make regular home visits to deliver the services	Home delivery	Anganwadi services	
Now everything has been back to normal	Return to normalcy	Health facility services	
Missed services are covered up	Covering up		

Medical officers mentioned the implementation of catch-up programs to address missed healthcare services. This included immunization catch-up initiatives and tracing back TB patients to ensure continuity of care. Plans were made to restart sterilization programs in the near future, aiming to cover services missed during the pandemic. Similarly, ASHA/ANMs reported covering missed services by cross-checking lists and providing vaccinations, with efforts made to ensure patients brought their children to hospitals.

Overall, catch-up programs had played a crucial role in addressing missed healthcare services and ensuring continuity of care for vulnerable groups post-COVID.

## 6 Discussion

This qualitative study shows the impact of COVID-19 on health services for vulnerable populations. We had considered people with health vulnerabilities like the elderly, antenatal mothers, children under 5 years, those with chronic diseases and immunocompromised conditions for the study. Single female household heads, pregnant and lactating mothers, persons with disabilities, older adult, and adolescents were described as the most vulnerable group during COVID-19 (18).

The study identified four main themes affecting the healthcare services during the pandemic period: (1) challenges faced and mitigation strategies adopted in accessing routine health services, (2) COVID-19 vaccination, (3) difference between the first and second waves of COVID-19, and (4) catch-up strategies used by the health system.

### 6.1 Challenges faced and mitigation strategies adopted in accessing routine health services

The patient-related challenges included difficulty in transportation, financial issues, anxiety, and attitude. A qualitative study conducted in Eastern India also found that financial issues and fear of COVID-19 infection were the major challenges faced by people with chronic morbidities (19). Similarly, a study done in six countries from Asia and Africa also identified changes in availability of services due to systems restructuring, difficulty affording care due to the economic impacts of the pandemic, and fear of contracting COVID-19 as the main barriers in accessing care during the pandemic (20). These identical findings from various studies across the low-middle-income countries shows the common nature of challenges faced in all similar settings.

Healthcare provider related challenges included inadequate protective measures, insufficient training, the diversion of resources to COVID-specific care, limitations on conducting physical examinations, and suspension of general OPD services. A mixed-methods study among 120 healthcare workers in India also found that healthcare facilities were affected and surgeries were postponed, particularly during the initial phase of COVID-19 (21). A rapid review identified that multiple factors, including fear of contracting the disease, lack of personal protective equipment, long working hours, quarantines, knowledge gaps, financial losses, staff shortages, and widespread community stigmatization, resulted in high levels of stress among healthcare workers (22). In a qualitative study conducted in Bangladesh, 15 in-depth interviews of healthcare workers revealed higher workload, psychological distress, shortage of quality personal protective equipment (PPE), social exclusion/stigmatization, lack of incentives, absence of coordination, and inadequate management during their service (23).

This evidence across settings consistently shows that the lack of preparedness and non-availability of personal protective equipment have significantly affected healthcare delivery during the pandemic. It underscores the need for integrating workforce protection, psychosocial support, and role clarity in to the epidemic preparedness plans. Unlike many previous narratives that highlight health workers as victims of pandemic-related stress, this study reflects on how they adopted protective behaviors and provided services without fear or stigma.

Health system-related challenges included staff shortages, lab closures, restricted hospital admissions, disruptions to immunization and other routine services, and delays in follow-up for chronic conditions. A cross-sectional study across six states in India yielded comparable findings. Lack of infrastructural preparedness during outbreaks, shortage of human resources, difficulty in outreach services and irregular supply of supplementary nutrition by Anganwadi workers were the main supply-side challenges identified (24). In a telephonic survey conducted among healthcare workers from 21 states in India, challenges were more evident in ensuring the availability of routine services and continuity of medications for non-communicable diseases (25). These observations indicate that the health system constraints identified in this study are in line with the national evidence, underscoring the consistent pattern of service disruptions across the country.

While similar service disruptions were reported in different settings, this study demonstrates how they were experienced simultaneously at the provider, health system, and beneficiary levels, resulting in a compounding effect across the continuum of care.

Mitigation strategies for addressing these challenges included adherence to COVID appropriate behavior in hospital premises, utilization of telemedicine, and home delivery of medicines for NCDs. A cross-sectional study from South India has also shown an increased adoption rate of telemedicine during the pandemic with 38.63% participants already using it (26). Similarly, a study from Thailand had identified telemedicine, community delivery of medicines, and decentralized care as innovations to sustain NCD services in the context of COVID-19 (27). These mitigation strategies played a crucial role in ensuring services during COVID-19 and highlight the need to incorporate them into preparedness frameworks for future public health emergencies.

### 6.2 COVID-19 vaccination

The challenges related to vaccination include complacency, confidence, and convenience (3 C's model); however, COVID vaccination represented a different scenario due to rapid research, accelerated vaccine development, and large-scale rollout (28). Apart from the usual 3 Cs, multiple additional challenges like hesitancy, availability, and registration issues were observed during the COVID-19 vaccination process.

Findings from the present study align closely with the existing literature, particularly regarding registration-related barriers. Online registration was one of the major challenges, especially in rural settings. Limited digital literacy, lack of identity cards, and a limited number of slots made it difficult to register for the vaccination. Similar issues were observed in qualitative studies done in India and Bangladesh. Community behavior, technical barriers, and dependence on digital platforms were found to limit vaccination uptake (29, 30). The reliance on E-mitra agents for registration emerged as a context-specific i.ssue in this study. The charges they collected imposed a financial burden on the community, creating a socioeconomic dimension to the “convenience” barrier described in existing literature.

The challenges in terms of availability, such as a lack of doses in rural areas, long waiting time, crowding at immunization centers, and the need to call the police to control crowd behavior, reported in this study, were similar to those identified in previous research conducted in other regions of India (31, 32). This study provides qualitative insights into how these availability constraints translated into operational challenges at the ground level.

Hesitancy emerged as a prominent challenge in this study. It was a result of fear of side effects, misinformation, concerns about vaccine safety, and misconceptions about COVID transmission among vaccinated individuals. These findings were consistent with earlier qualitative studies that identified fear, misbelief, and the influence of negative information as key drivers of vaccine hesitancy (29, 31). Elements of complacency were also evident, particularly among villagers who perceived low risk due to restricted mobility and absence of cases in their locality.

The present study also highlights the enabling factors that facilitated vaccination uptake. Doctor-led counseling, involvement of local leaders, community outreach, spot registration, deployment of dedicated staff, and home-based vaccination for bedridden individuals played a critical role in addressing hesitancy and access barriers. Similar findings were observed in a qualitative study done in Maharashtra, India, underscoring the importance of community-centered and health system driven approaches for achieving improved vaccination coverage (31).

The consistency of findings across different studies and settings suggests that the challenges encountered during COVID-19 vaccination were systemic rather than isolated. Lessons learned from the COVID-19 vaccination experience should be used as a reference for future outbreaks requiring rapid vaccine development or mass roll out. The findings from this study emphasize the importance of moving beyond the traditional 3 Cs model to ensure the success of vaccination campaigns.

### 6.3 Difference between the two waves

The present study has considered March-August 2020 as the first wave and March-August 2021 as the second wave of COVID-19. Healthcare service delivery across the two waves was dependent on multiple factors, including disease severity, system readiness, and community attitudes. During the first wave, fear had led to compliance with preventive measures but health services were significantly disrupted. While in the more severe second wave, reduced fear resulted in non-compliance to preventive measures but there was marked improvement in the service continuity.

Consistent with findings from multiple hospital-based studies across India and other countries, the second wave emerged as the most severe phase of the pandemic. Studies from different settings in India- Rajasthan, Maharashtra, Delhi, and Rishikesh have uniformly reported higher hospitalization rates, greater need for oxygen and ventilatory support, increased ICU admissions, and significantly higher mortality during the second wave compared to the first (33–37). Similar findings were also observed in a study done in Brazil, which documented increased invasive respiratory support and more severe clinical manifestations during the second wave (38).

The findings from the present study complement and help explain the patterns observed in other studies which were largely quantitative studies. By considering the interplay of disease severity, health system preparedness, and community attitudes, it provides deeper insight into why the differences manifested from both health system and community perspectives. This helps in understanding how differences between waves translate into variations in healthcare service delivery, particularly for vulnerable populations.

### 6.4 Catch-up programs

The findings from this study underscore the need for catch-up programs to restore healthcare services post-disruption. In Jodhpur, strategies such as home-based service delivery, list-based cross-verifications, immunization catch-up sessions, and tracing back tuberculosis patients were used after the pandemic. Studies examining the catch-up strategies used in post-disruption contexts remain limited, underscoring the significance of these qualitative insights for uncovering service restoration beyond emergency response.

Comparable findings have been reported in few other studies. A study done in Rajasthan pointed out that the state had resumed immunization services quickly through outreach activities but did not fully cover for the doses missed during lockdown, particularly among underserved groups (39). A qualitative study from Madhya Pradesh, India, also documented complete disruption of immunization during the pandemic. Catch-up strategy planned in two phases, emphasizing on awareness campaigns including door-to-door health education, expanded immunization days to reduce crowding, and strengthened infection-prevention measures, resulted in successful coverage of most left-out children (40). Similarly, a study done in Kerala, India, reported the use of a targeted approach to maintain the continuity of TB-related health care services (41). Collectively, these findings reinforce the need for catch-up programs to ensure the equitable recovery of health care services post-disruptions, particularly among vulnerable groups. Recovery should be considered as an active phase in the emergency cycle, requiring as much planning and resourcing as the response phase itself. Successful recovery will depend on the effective coordination, community engagement, and frontline worker initiatives.

By bringing together these four interconnected dimensions, this study underscores the need for a preparedness framework inclusive of vulnerable groups. This framework must ensure the continuity of essential services, protect the healthcare workforce, incorporate structured recovery planning, and facilitate strong coordination across health and allied sectors during future outbreaks.

## 7 Limitations

A purposive sampling strategy limits the generalizability of the results beyond the study setting. The interviews were conducted within health facility settings, which may have introduced social desirability bias, particularly among beneficiaries who could have been reluctant to express dissatisfaction with services received. The inclusion of heterogeneous categories of vulnerable population, such as pregnant women, elderly individuals, and patients with chronic conditions, to capture health system level challenges from different perspectives; condition-specific experiences may not have been sufficiently explored.

## 8 Conclusion

The COVID-19 pandemic disrupted the routine health services, including national health programs. The initial phase of lockdown and subsequent waves posed significant challenges for vulnerable populations in accessing routine healthcare services, exacerbating existing health disparities. Factors such as limited access to healthcare facilities, financial constraints, transportation difficulties, and fear of contracting the virus deterred many from accessing necessary medical services. The increased vulnerability of high-risk groups was evident from past outbreaks, yet there were still noticeable gaps in delivering healthcare services to these populations during the COVID-19 pandemic.

This study underscores the critical importance of addressing the specific needs of vulnerable populations during any public health crises. By implementing targeted support interventions which are culturally sensitive and linguistically appropriate, healthcare providers can effectively reach and support vulnerable communities, bridging gaps in healthcare access and addressing disparities in health outcomes.

By prioritizing the needs of vulnerable populations and strengthening collaboration between healthcare providers, community organizations, and policymakers, societies can enhance preparedness and responsiveness in future crises. It is vital to ensure equitable access to care and safeguard the health and well being of all individuals, particularly those at higher risk.

## Data Availability

The original contributions presented in the study are included in the article/supplementary material, further inquiries can be directed to the corresponding authors.
